# Role of Computed Tomography in the Evaluation of Peritoneal Carcinomatosis

**DOI:** 10.5334/jbsr.2921

**Published:** 2023-04-11

**Authors:** Snehal Kose

**Affiliations:** 1 All India Institute of Medical Sciences, New Delhi, IN

**Keywords:** peritoneal carcinomatosis, heated intraperitoneal chemotherapy (HIPEC), early post operative chemotherapy (EPIC), computed tomography

## Abstract

Peritoneal carcinomatosis (PC) refers to metastatic spread of tumor into the peritoneal cavity. Earlier, PC was thought to be associated with grave clinical outcome. However, various advances in treatment options including cytoreductive surgery and heated intraperitoneal chemotherapy or early post-operative chemotherapy can prolong survival of patients with peritoneal carcinomatosis. These treatment options are associated with high morbidity and mortality. The purpose of this article is to acquaint the radiologist about various appearances of peritoneal carcinomatosis in order to help clinicians in selecting candidates for surgery and avoid unnecessary potentially debilitating surgeries in patients with unresectable PC.

## Introduction

Peritoneal carcinomatosis refers to spread of malignancy along peritoneal lining. Earlier peritoneal carcinomatosis was considered to carry grave clinical prognosis with short survival life post diagnosis. Nowadays, many therapies like cytoreductive surgery (CRS) and heated intraperitoneal chemotherapy (HIPEC) or early post-operative chemotherapy (EPIC) are available for treatment of PC. However, these surgeries are associated with high mortality and morbidity and hence should be done only in patients who are appropriate candidates for surgery in whom surgery is expected to increase survival. Patients with unfavourable sites of involvement by PC should be identified so that potentially debilitating surgeries can be avoided in these patients.

## Anatomy

The peritoneum is the largest serous membrane covering the abdominal cavity. It consists of two layers, the outer parietal layer, which lines the abdominal cavity and pelvis, and the inner visceral layer, which covers intraperitoneal visceral organs. Several folds of visceral peritoneum suspend the visceral organs in abdominal cavity and divide the abdomen into various compartments.

### Supramesocolic (SM) compartment

**Figure 1:**
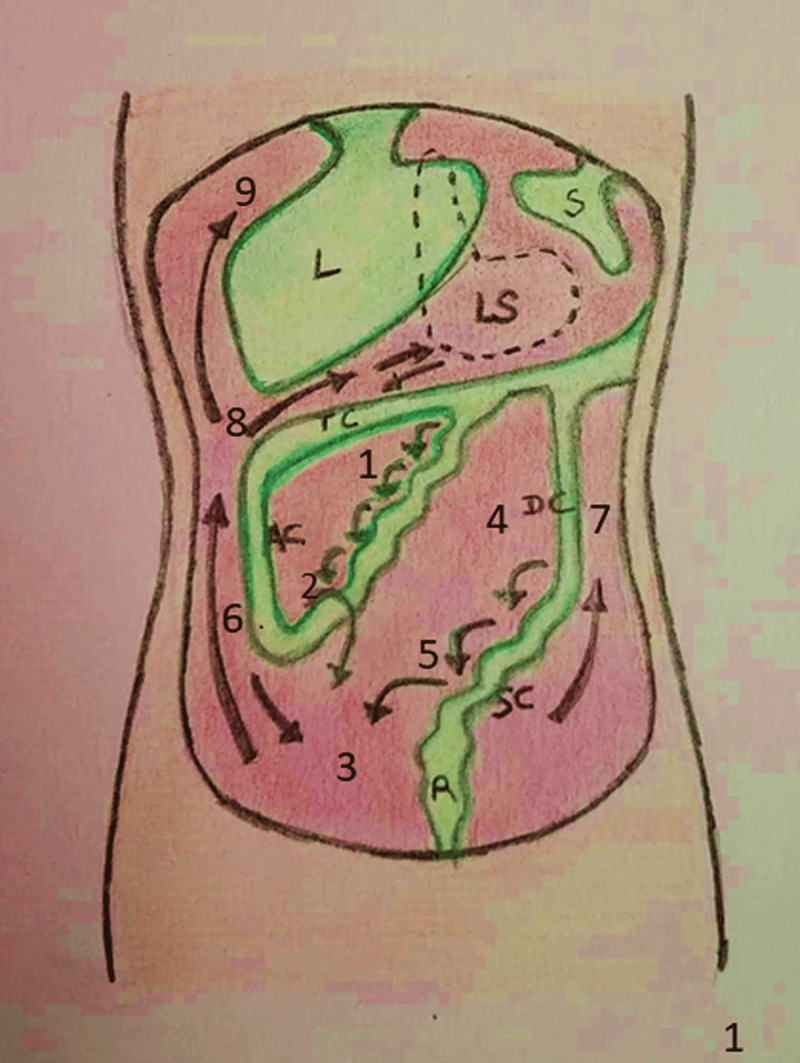
Pathway for flow of peritoneal fluid. Peritoneal fluid from right inframesocolic compartment (1) collects in right iliac fossa near ileo-cecal junction (2) which later drains into pelvis (3). Peritoneal fluid in left inframesocolic compartment (4) is collected at surface of sigmoid colon (5) and later drains into pelvis. Fluid from pelvis is drawn upwards in right paracolic gutter (6), right subhepatic (8) and subphrenic spaces (9) by diaphragmatic movement. Upward flow of fluid from left paracolic gutter is prevented by phrenicocolic ligament.

The abdominal cavity is divided into supra and inframesocolic compartments (Figure 1) by transverse mesocolon [1,2]. The supramesocolic compartment (space above transverse mesocolon) is divided into right and left parts by the falciform ligament [3]. The right SM compartment consists of right subphrenic space (bounded anterosuperiorly by right hemidiaphragm, inferiorly by right lobe of liver and medially by falciform ligament), right subhepatic space, and lesser sac. The right subhepatic space is further divided into anterior and posterior (Morisson’s pouch) compartments. The lesser sac is situated between stomach anteriorly and pancreas posteriorly [4]. It communicates with the peritoneal cavity through foramen of Winslow.

The left supramesocolic compartment consists of perihepatic and subphrenic spaces. Perihepatic space is divided into anterior (between diaphragm anterosuperiorly and left lobe of liver posteriorly) and posterior (posterior to left lobe of liver and anterior to stomach and gastrohepatic ligament) compartments. The left subphrenic space is divided into anterior part (just lateral to left lobe of liver) and posterior (perisplenic) compartments [5].

### Inframesocolic compartment

It is divided into right and left parts by mesentery which extends from ligament of Treitz to ileo-cecal junction. Inframesocolic compartment consists of right and left paracolic gutters (spaces lateral to ascending and descending colon respectively) and mesentery. The right paracolic gutter is wider and communicates freely with the right SM compartment while the left paracolic gutter is shallow and separated from the left SM compartment by phrenicocolic ligament [6]. In males the peritoneum forms a closed sac showing no communication with pelvis, while in females the peritoneum is perforated by fallopian tubes resulting in free communication between abdominal and pelvic cavities [7].

## Sites of Lodgement of Metastases

The potential space between parietal and visceral layers of peritoneum is called the peritoneal cavity and is filled with a small amount of peritoneal fluid, which allows frictionless movement of visceral organs within the abdominal cavity.

There is a distinct pattern of flow of peritoneal fluid in abdomen (Figure 1) [2]. In upright position, peritoneal fluid accumulates in most dependent portions of abdomen including rectouterine/rectovesical and paravesical pouches. Fluid in the right inframesocolic compartment flows towards the ileocecal junction, where it accumulates temporarily, while in the left inframesocolic compartment, it flows towards the surface of the sigmoid colon. With expiration, the diaphragm moves upwards, resulting in negative intra-abdominal pressure, which draws fluid in the cephalad direction from the right paracolic gutter to the right subhepatic and the subphrenic spaces. Peritoneal fluid resorption takes place at greater momentum and right subphrenic space [8,9]. Metastases occur at sites of stasis and absorption of peritoneal fluid (Table 1).

**Table 1:** Common sites for peritoneal implants.


AREAS OF STASIS OF PERITONEAL FLUID FLOW	AREAS OF FLUID RESORPTION

Rectouterine/rectovesical space	Greater omentum

Right lower quadrant—ileocecal region	Right subdiaphragmatic space

Left lower quadrant—superior aspect of sigmoid	

Right paracolic gutter	

Pouch of Morrison	


## Routes of Spread of Peritoneal Malignancies [10]

- **Direct spread:** Tumours can spread by direct invasion of peritoneum and mesentery. GI tract and pancreatic malignancies spread by this route [11].
- **Hematogenous spread:** Lung, breast cancer, and melanomas can spread by this route. The metastatic deposits are usually located along antimesenteric border of small bowel in the case of hematogenous seeding.
- **Lymphatic spread:** Lymphomas and various malignancies can spread through lymphatics with resultant enlarged lymph nodes/lymph nodal masses.
- **Peritoneal seeding**

## Imaging in Peritoneal Carcinomatosis

### Mdct

MDCT of the thorax and abdomen following oral positive and intravenously administered contrast in the current modality of choice for suspected peritoneal carcinomatosis. Oral positive contrast helps in detection of hypodense serosal deposits on the bowel surface, but calcified deposits and subtle enhancing bowel wall thickening can be missed [12]. A scan is performed in the portal venous phase (60–70 seconds post contrast administration). Computed tomography (CT) is considered the imaging modality of choice due to easy availability, lower cost, high spatial resolution, lesser scan time, and multiplanar reconstruction.

**Figure 2:**
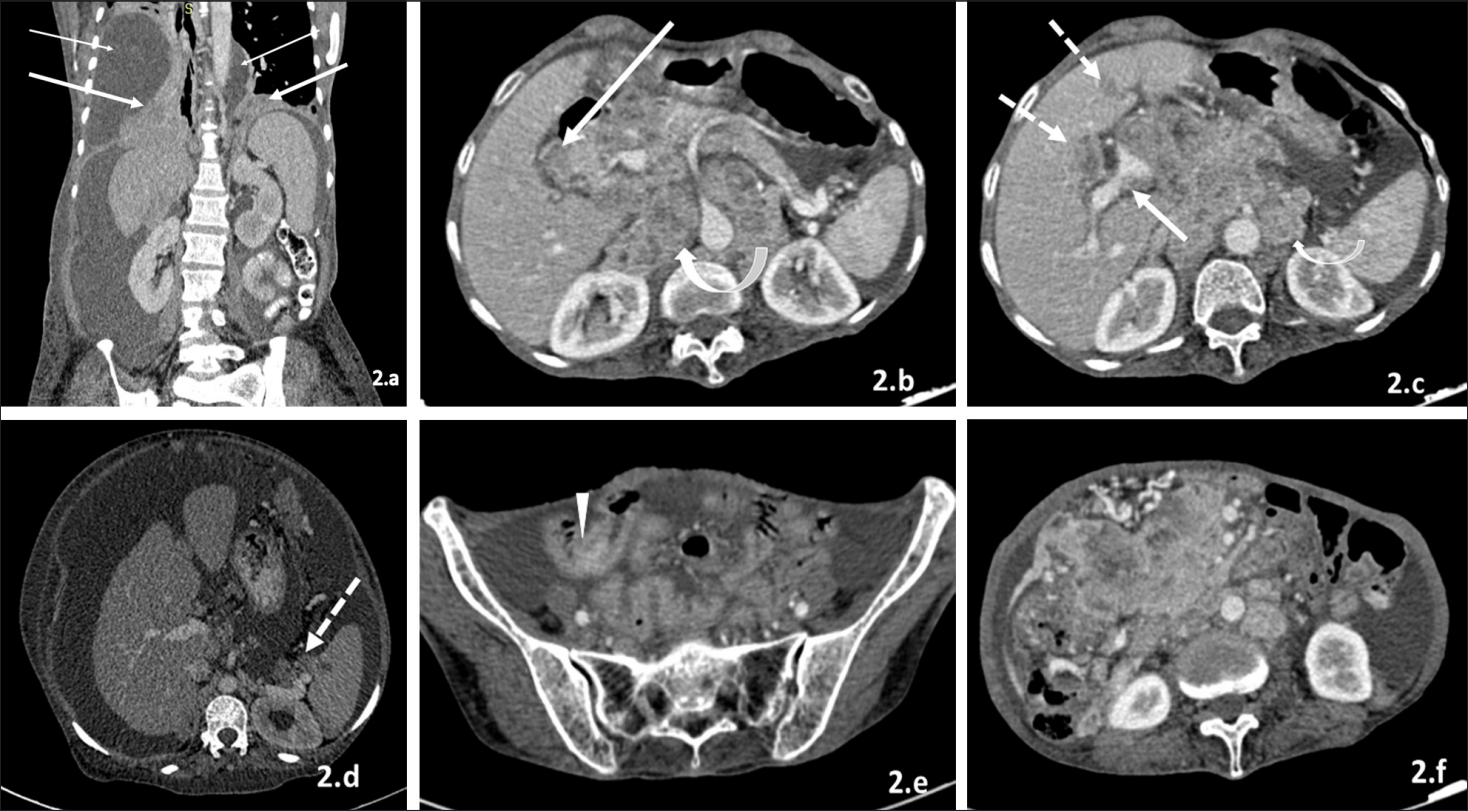
**(a–f)** Inoperable disease. 1) Extraperitoneal spread: pleural effusion (thin white arrows in a), pleural deposits (thick white arrows in a), retroperitoneal lymph nodes (curved white arrows in b and c). 2) Intraparenchymal deposits (dotted arrows in c). 3) Hepatic hilum (thick arrow in b) and splenic hilar deposits (dotted arrow in d). 4) Wall thickening of bowel loops with or without obstruction (arrowhead in e). 5) Deposits adjacent to aorta, IVC (b and c), or other major vessels (solid arrow in c). 6) Extensive involvement of mesentery (f).

The various sites and patterns that must be looked for in CT scan in suspected case of PC are as follows:

**1)** Extraperitoneal disease – including pleural effusion, pleural thickening, nodules, lung metastases, and retroperitoneal lymph nodes/masses (Figure 2). Presence of extraperitoneal disease is considered contraindication for CRS [13].

**2)** Surface deposits – on solid organ (liver, spleen, kidney) surface. They appear as nodules/plaques/biconvex masses with or without scalloping of liver surface (Figure 2 a).
**3)** Intraparenchymal deposits – in visceral organs can appear as well-/ill-defined hypodense nodules with or without peripheral enhancement on arterial phase which become homogenously hypodense on venous phase (Figure 2c). Deposits from hypervascular tumours, such as neuroendocrine tumours, melanoma, and so on, appear hyperenhancing on arterial phase.
**4)** Peritoneal ligaments – deposits appear as enhancing nodules/plaques/masses at sites of ligaments.

**Figure 3:**
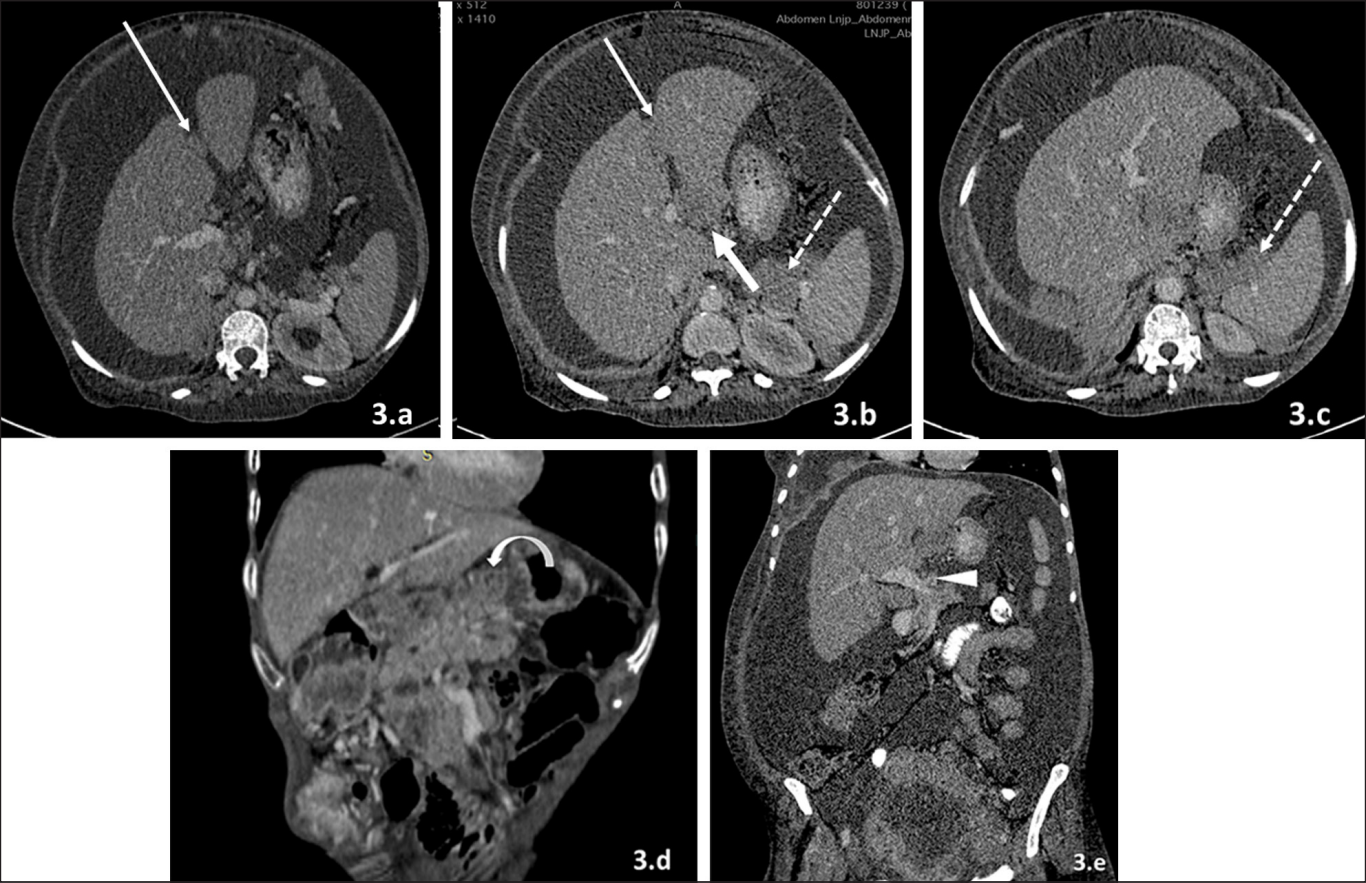
**(a–e)** Deposits along peritoneal ligaments. A) Falciform ligament/ligamentum teres: CECT image of the abdomen in a case of ovarian carcinoma reveal heterogeneously enhancing soft tissue deposits along ligamentum teres/falciform ligament (thin arrow in a and b). B) Ligamentum venousum: CECT image of the abdomen in a case of ovarian carcinoma reveal heterogeneously enhancing soft tissue deposits along ligamentum venosum (thick arrow in b). C) Gastrohepatic ligament: CECT images of the abdomen in a case of breast carcinoma reveal heterogeneously enhancing soft tissue deposits in gastrohepatic ligament between left lobe of liver and lesser curvature (curved arrow in d). D) Hepatoduodenal ligament: CECT image of the abdomen in a case of ovarian carcinoma reveal ill-defined soft tissue mass along portal vein reaching up to porta hepatis (arrowhead in e). E) Gastrosplenic ligament: CECT image of the abdomen in a case of ovarian carcinoma reveal heterogeneously enhancing soft tissue deposit along gastrosplenic ligament (dotted arrows in b and c).

The various peritoneal ligaments and fissures, which can serve as sites for lodgement of metastatic deposits, include:

**a)** Perihepatic fissures/ligaments (Figure 3) including falciform ligament/ligamentum teres (which separates medial and lateral segments of left lobe), gall bladder fissure (which separates right and left lobes of liver) and ligamentum venosum (which separates caudate lobe from left lobe). All these ligaments are connected to porta hepatis and deposits along these fissures/ligaments can spread to porta hepatis leading to fat porta sign [14].

**Figure 4:**
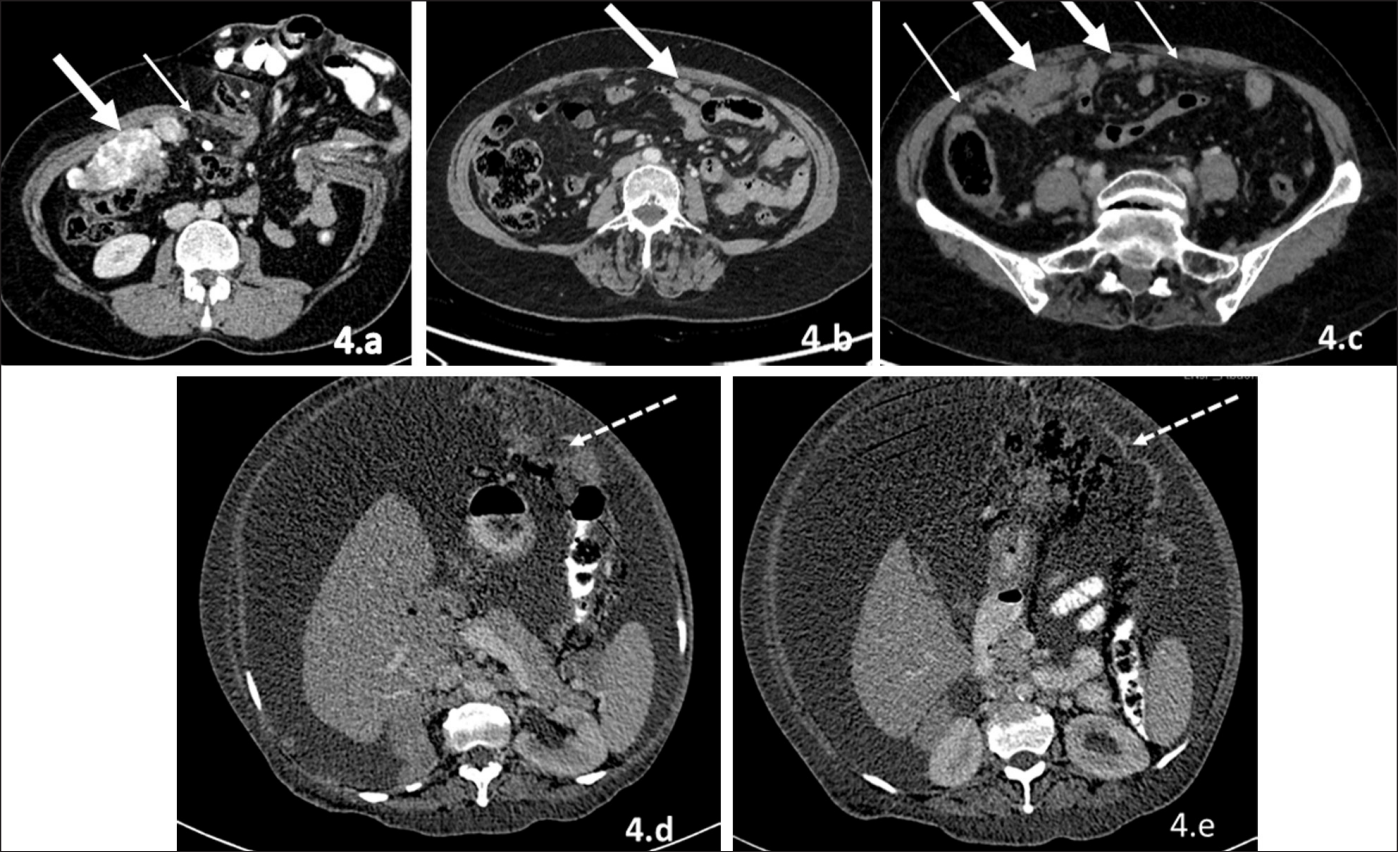
**(a–e)** Patterns of involvement of omentum by peritoneal carcinomatosis. 1) Diffuse stranding: CECT images of the abdomen reveal omental fat stranding (thin arrows in a and c) along with soft tissue deposits. 2) Nodularity and masses: CECT images of the abdomen reveal soft tissue nodules and masses in greater omentum (thick white arrows in a, b, and c). Soft tissue deposits in figure a are calcified (metastases from mucinous adenocarcinoma of colon). 3) Sheet-like masses (omental caking): dotted arrows in d and e.

**b)** Lesser omentum (gastrohepatic ligament): It connects the left lobe of the liver with lesser curvature of stomach. Gastric cancer can spread along the liver surface by this route. It is also connected to the hepatoduodenal ligament, which serves as transport medium for pancreatic cancer along liver surface, porta hepatis, and stomach (Figure 3d).
**c)** Hepatoduodenal ligament: It connects the porta hepatis to the duodenum and contains the main portal vein, common bile duct, and hepatic artery. It serves as conduit for the spread of pancreatic cancer (Figure 3e).
**d)** Greater omentum (Figure 4): It connects the greater curvature of stomach with the transverse mesocolon. Deposits along the greater omentum can appear as diffuse stranding of omentum, nodules or masses within omentum or sheet like masses within omentum called as omental caking.

**e)** Gastrosplenic ligament (Figure 3b and 3c): It is present between the greater curvature of the stomach and spleen. The lineorenal ligament connects the spleen to the left kidney and lodges the pancreatic tail. Deposits can also be lodged along these ligaments.

**Figure 5:**
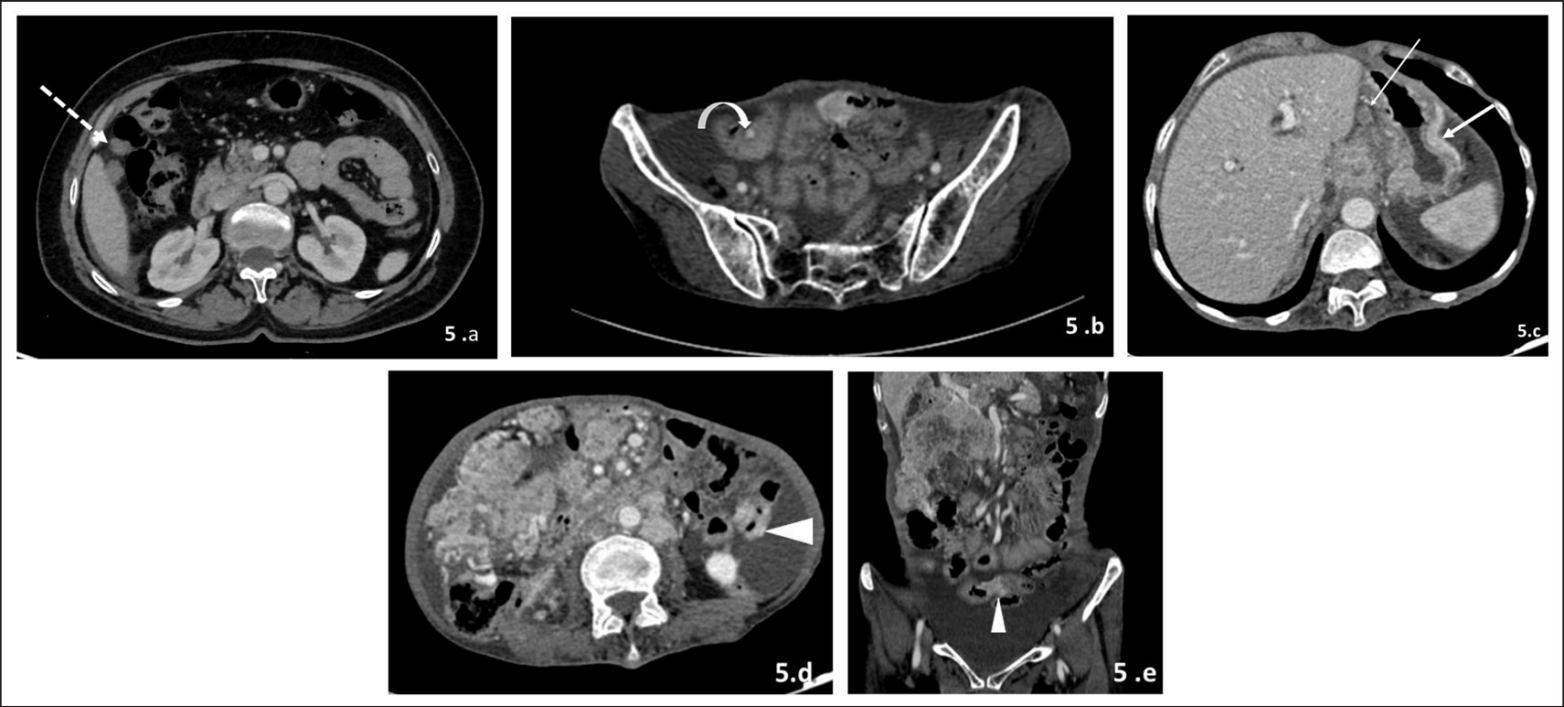
**(a–e)** Patterns of deposits involving gastrointestinal tract. 1) Nodules/masses: CECT image of the abdomen (a) reveals soft tissue deposit along hepatic flexure of colon (dotted arrow). Lower sections of the abdomen in the same patient small submucosal nodule in ileal loop (curved arrow in b). 2) Sheet like masses: CECT image of the abdomen (c) reveals enhancing sheet like soft tissue mass along greater curvature of stomach (thick arrow). Few necrotic deposits are also seen along gastrohepatic ligament (thin arrow). 3) Wall thickening and enhancement: CECT images of the abdomen (d and e) reveal focal enhancing wall thickening along walls of small bowel loops with resultant mild luminal narrowing (arrowhead).

**5)** Serosal deposits (Figure 5): Serosal deposits along the surface of the gastrointestinal tract can appear as single or multiple nodules, masses, diffuse sheet-like masses over the surface, masses contiguous with adjacent mesentery, or subtle wall thickening and enhancement, which can lead to intussusception or bowel obstruction. These are particularly common in the ileocecal junction and the sigmoid colon, where stasis of the peritoneal fluid takes place.

**Figure 6:**
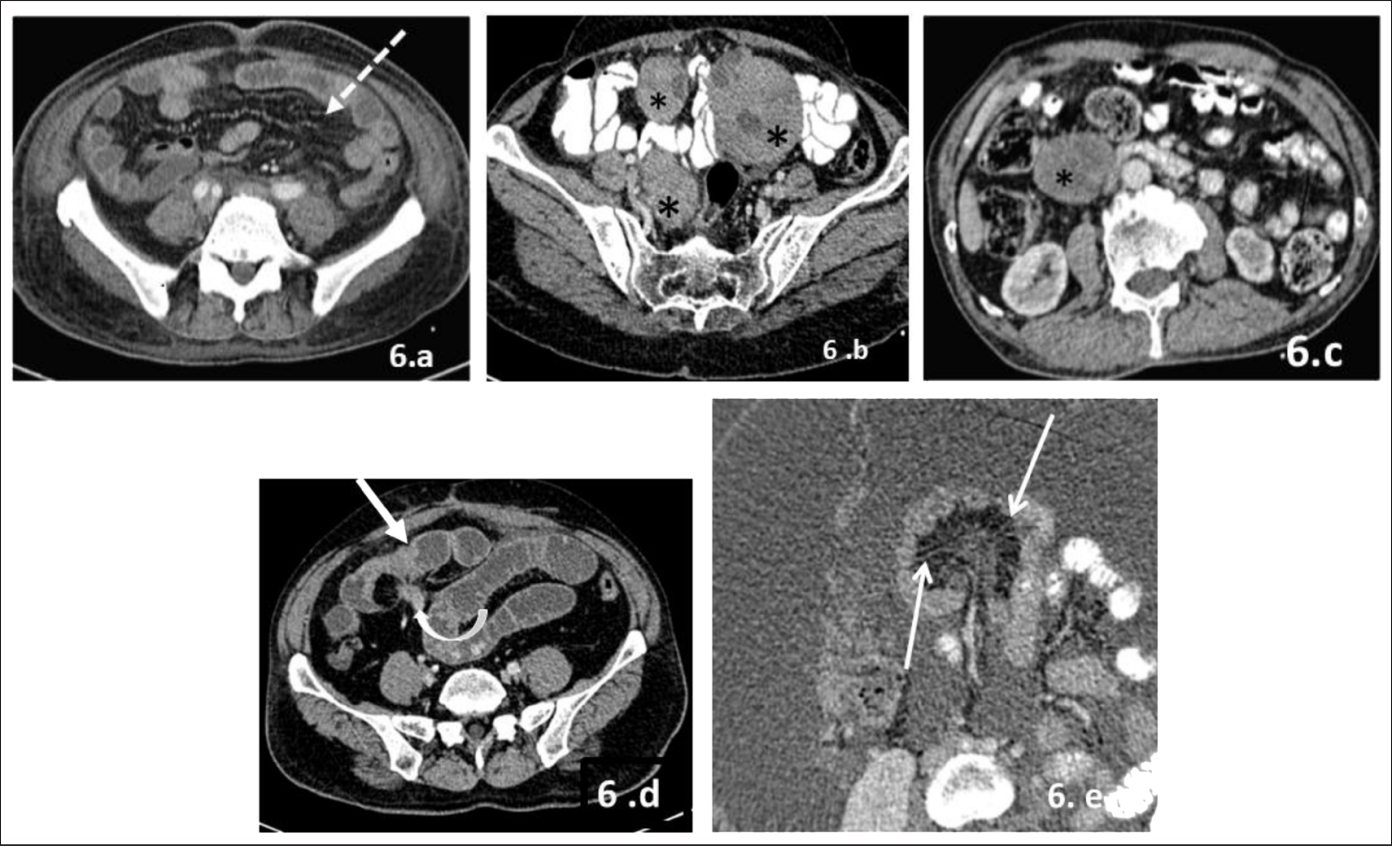
**(a–e)** Patterns of involvement of mesentery in peritoneal carcinomatosis. 1) Diffuse mesenteric fat stranding (dotted arrows in a). 2) Nodules/masses: CECT images of the abdomen in patient with disseminated granulosa cell tumour of ovaries (b) reveal multiple solid cystic masses in mesentery (asterisk). CT scan in another patient with bronchogenic carcinoma (c) reveal soft tissue deposit in mesentery (asterisk). 3) Mass contiguous with small bowel thickening/mass: CECT image of the abdomen in a patient with ileal carcinoid reveal ill-defined enhancing wall thickening in terminal ileal loops along with spiculated mass in adjacent mesentery (d). 4) Stellate mesentery: Axial contrast enhanced CT images (e) of the abdomen in a patient with ovarian carcinoma reveal gross ascites, ill-defined stranding in mesentery along mesenteric vessels resulting in thickening and rigidity of mesentery and straightening of mesenteric vessels (thin white arrows).

**6)** Mesentery (Figure 6): Mesentery is a fan-shaped fold of peritoneum that suspends loops of small bowel from the posterior abdominal wall and extends from the duodenojejunal junction to the ileo-cecal junction in the right lower quadrant. Mesenteric involvement by metastases can appear as diffuse stranding, multiple nodules, focal masses, or masses contiguous with bowel serosa. Sometimes, stellate or frozen mesentery can be seen on imaging which refers to ill-defined soft tissue thickening along mesenteric vessels with straightened and stretched mesenteric vessels.

**Figure 7:**
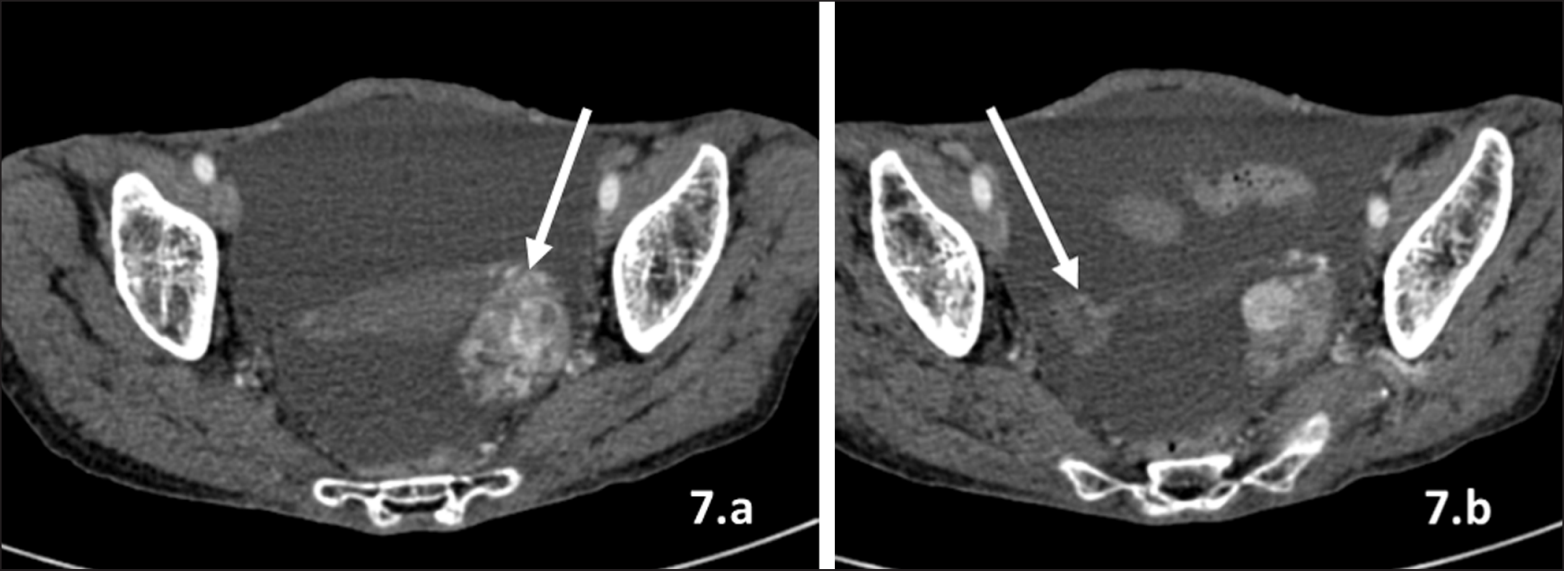
**(a–b)** Krukenberg tumour. Axial contrast-enhanced CT images of the abdomen in a post operative case of breast carcinoma reveal smooth-marginated solid cystic mass lesions in bilateral ovaries (white arrows) and ascites.

**7)** Pelvis (Figure 7): Metastatic deposits tend to occur at the sites of stasis of peritoneal fluid in the pelvis including the rectouterine, rectovesical, vesicouterine, and paravesical pouches, along the surface of fallopian tubes, broad ligaments, and ovary. Krukenberg tumours are serosal deposits on the ovary from adenocarcinomas, most commonly signet ring cell carcinomas of the stomach and breast and colorectal carcinomas. They usually appear as bilateral solid cystic masses with smooth surface in bilateral ovaries.

**Figure 8:**
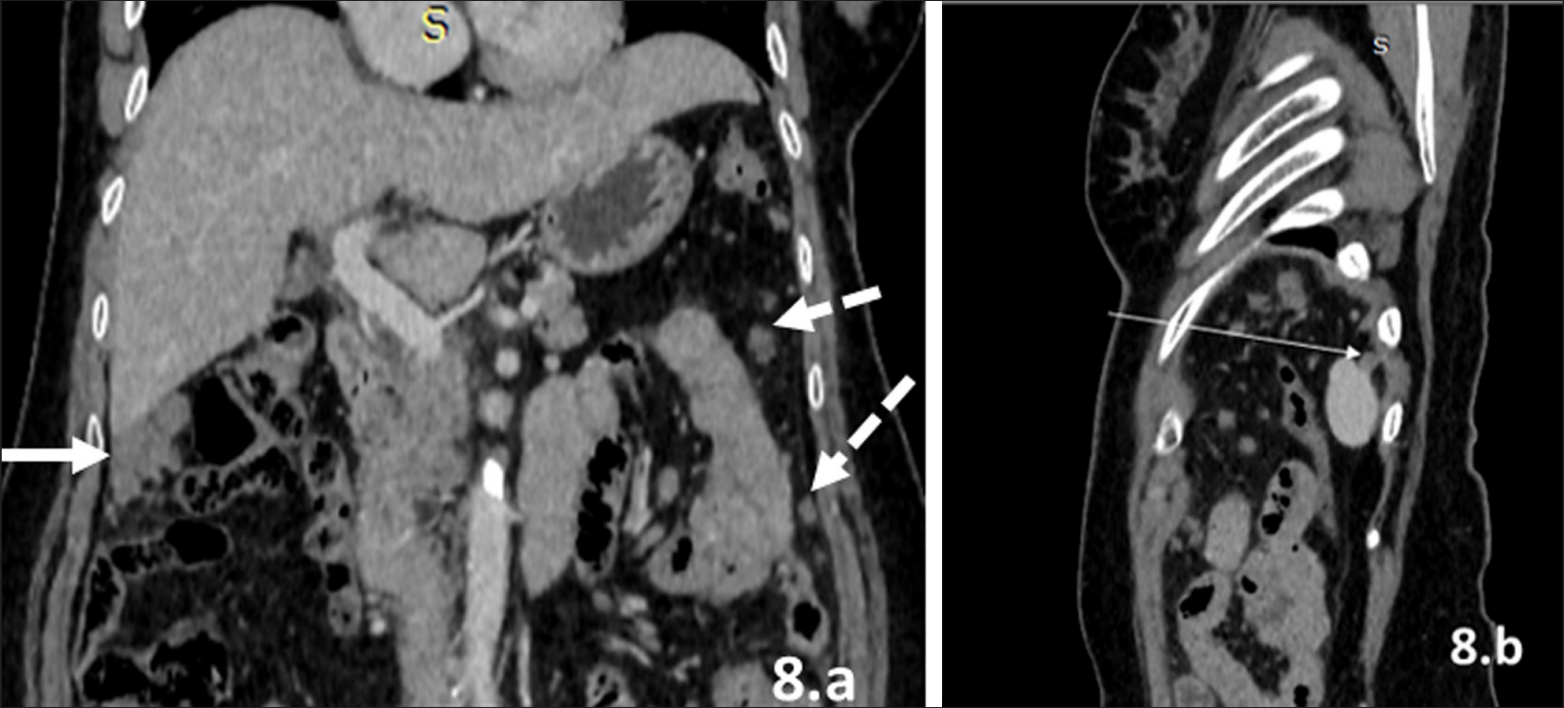
**(a–b)** Regions of stasis of peritoneal fluid. CECT scan of the abdomen in a case of mucinous cystadenocarcinoma of ovary reveal soft tissue deposits in right subhepatic space (thick white arrow), left paracolic gutter (dotted white arrow), and left subphrenic space (thin white arrow).

**8)** Peritoneal lining: Usually the peritoneal lining is not visible or seen as a discontinuous line on CT. In PC, peritoneal involvement appears as continuous peritoneal thickening, irregularity, and enhancement, nodules, or masses. Ascites can be seen.
**9)** Sites of stasis of peritoneal fluid (Figure 8): These sites include the bilateral paracolic gutters, bilateral subphrenic spaces, right subhepatic space, and perihepatic spaces.

However, CT may fail to detect small (<5 mm) deposits and deposits in specific sites such as the right subphrenic space, lesser sac or mesenteric root, and serosal deposits [14,15,16,17,18]. Kim et al. found sensitivity and specificity of CT for detecting peritoneal deposits to be 89% and 65% respectively [19].

### Mri

MRI is usually used in cases where CT is inadequate/doubtful for presence of metastases [20,21,22]. MRI performs better than CT for detection and characterization of metastatic deposits due to higher soft tissue contrast resolution. Metastatic deposits appear hypointense on T1WI and heterogeneously hyperintense on T2WI. Moreover, newer sequences like DWI can detect small (< 5mm) metastatic deposits to peritoneum as well as deposits in sites where CT proves inadequate including subphrenic spaces, lesser omentum, serosal, and mesenteric deposits [23]. Dynamic contrast-enhanced MRI can show delayed enhancement of involved peritoneal lining. MR spectroscopy has not been much investigated in PC; however, studies have been done that revealed increased choline in metastatic deposits. Yu et al. found sensitivity and specificity of MRI for detecting peritoneal deposits in ovarian malignancy to be 88% and 99% respectively [24].

However, MRI is not routinely used to due to cost issues and prolonged scan time.

### Usg

USG can detect intraparenchymal deposits, surface deposits, ascites, and larges deposits in mesentery; however, complete evaluation of abdominal cavity is often inadequate. Nowadays, USG is reserved for percutaneous biopsy of lesions on real-time imaging [10,15].

### FDG-PET-CT

FDG PET-CT is more accurate than FDG-PET or CT alone and gives functional as well as anatomic information. Lesions not well delineated on CT are better assessed on PET-CT, which shows FDG-uptake due to increased glucose metabolism in malignant tissues. Metabolic tumor burden measures derived from PET-CT, namely metabolic tumour volume (MTV) and total lesion glycolysis (TLG) offer prognostic information in peritoneal carcinomatosis. However, diffuse extensive peritoneal deposits show mild uptake on PET-CT, an inherent limitation of PET-CT in peritoneal malignancies [25,26]. False negative PET-CT results can be seen in case of small deposits, mucinous adenocarcinomas, and neuroendocrine tumours which show no/minimal FDG uptake [26,27,28,29], and false positive results can be seen in infectious and inflammatory conditions like tuberculosis [30]. In a study conducted by Satoh et al., sensitivity and specificity of 94% have been reported for PET-CT for detection of peritoneal carcinomatosis of ovarian primary [31]. PET-CT has been found superior to CT and MRI in detecting peritoneal metastases in studies undertaken by Kim [19] and Yu et al. [24] as well.

## Role of Imaging in Patient Management

Earlier, peritoneal carcinomatosis was managed by palliative chemotherapy. Various advancements in treatment strategy of PC have been developed over years which include peritonectomy and visceral resections. In a study conducted by Elias et al., median survival was observed to be 23.9 months in the standard group receiving systemic chemotherapy versus 62.7 months in the patients who underwent CRS and HIPEC [32]. The various surgeries used to be performed for peritoneal carcinomatosis [33] are listed in Table 2. Nowadays, patients with PC are managed with cytoreductive surgery (which refers to resection of macroscopic metastatic disease) with HIPEC (heated intraperitoneal chemotherapy) or EPIC (early intraperitoneal chemotherapy), which has been proven to increase survival in these patients [34,35,36]. This is a potentially debilitating surgery with high post-operative morbidity and mortality [37,38] and hence selection of appropriate candidates for this surgery is important as it can prolong survival in patients where complete cytoreduction can be achieved and avoid surgery in non-suitable candidates. Various criteria for selection of appropriate candidates include clinical criteria (ECOG performance status < 2) [39], histological criteria (favourable outcome in disseminated peritoneal mucinosis and node negative colorectal carcinoma [34,40] while worst outcome in gastric adenocarcinoma and malignant mesothelioma) [41] and radiological criteria.

**Table 2:** Peritonectomy and resections used to achieve cytoreduction.


PERITONECTOMY	RESECTIONS

Anterior parietal peritonectomy	Old abdominal incisions and umbilicus

Left upper quadrant peritonectomy	Greater omentectomy and spleen

Right upper quadrant peritonectomy	Tumour on Glisson capsule of the liver

Left parietal peritonectomy	Uterus, ovaries, and rectosigmoid colon

Right parietal peritonectomy	Gall bladder and lesser momentum

Pelvic peritonectomy	

Omental bursectomy	


An important criteria to be mentioned in the radiological report is the peritoneal carcinomatosis index (PCI). This index was given by Sugarbacker et al. and is a measure of evaluation of volume and extent of peritoneal carcinomatosis [42,43]. It is calculated intraoperatively but can be calculated preoperatively on CT. CT-based PCI has been proved as accurate as intraoperative PCI [44]. A recent study by Flicek et al. [45] reported a moderately good correlation between the radiologic PCI score and the surgical PCI score (sensitivity, 76%; specificity, 69%). The use of radiological PCI score facilitates the communication between radiologists and surgeons, and it could be useful for surgical planning [33,46]. In this, the abdomen is divided into nine compartments with additional four segments of small bowel (upper jejunum, lower jejunum, upper ileum, and mid ileum). Lesion size is determined by longest dimension and given four scores:

LS0- No intraperitoneal disease

LS1- Lesion size < 0.5 cm

LS2- Lesion size between 0.5 to 5 cm

LS3- Lesion size > 5 cm, confluent mass/caking

Scores in 13 parts of the peritoneal cavity is summed up to give PCI, which ranges from 0 to 39. A PCI score greater than 20 generally precludes complete cytoreduction. Moreover, involvement of jejunal regions 9 and 10 has more unfavorable prognosis than involvement of ileal regions 11 and 12 [47,48]. In a study conducted by Dohan and coworkers, PCI obtained using both CT and MRI was more accurate in predicting the surgical PCI than CT alone [49], while in a study conducted by Low et al., MRI alone was more more predictive of surgical PCI than CT-PCI [50].

## Validity of PCI in Various Cancers

While planning for CRS, the tumor entity should also be taken into account, along with PCI. For example, patients with peritoneal carcinomatosis of colonic origin with a PCI ≤ 20 qualify for CRS and HIPEC while the PCI in patients with gastric cancer should be < 10 or ≤15 [51,52].

In patients with pseudomyxoma peritonei arising from mucinous neoplasms PCI > 20 is no absolute exclusion criteria since these patients have good prognosis despite high tumour load. In these patients, tumour grading, extent of mesenteric invasion, liver metastasis, and age play an important role in conjunction with PCI [52]. For patients with colorectal cancer and peritoneal disease, there is a selection criterion that combines various prognostic factors like PCI score, tumour differentiation, and patient symptoms, into a scoring system known as the peritoneal surface disease severity score [53].

## Structured Reporting in a Case of Peritoneal Carcinomatosis

1. Presence of extraperitoneal disease (pleural effusion, lung metastases, retroperitoneal lymph nodes/masses)
2. Presence of intraparenchymal deposits
3. Presence of hepatic or splenic hilar deposits
4. Number, site, and size of peritoneal deposits (serosal, peritoneal spaces, along ligaments, mesentery, and omentum)
5. Presence of ascites, peritoneal thickening, enhancement, and irregularity
6. Presence of deposits > 5 cm in longest dimension
7. Presence of bowel wall thickening with or without obstruction
8. Presence of ureteric involvement (focal enhancing wall thickening/masses)
9. Presence of deposits adjacent to IVC, aorta, or other major vessels
10. Presence of deposits in mesenteric root/involving proximal aspect of celiac trunk, SMA, SMV, and IMA. Presence of extensive mesenteric involvement in the form of stellate mesentery/diffuse nodularity/root involvement.
11. Abdominal/pelvic wall involvement
12. Calculation of CT-PCI
13. Final comment if the disease is resectable/resectable with increased surgical complexity/unresectable
14. Recommendation of additional imaging (MRI/PET-CT) when in doubt.

## Management

### Types of Surgeries

In the early 1990s, Sugarbaker et al. introduced cytoreductive surgery (CRS) and hyperthermic intraperitoneal chemotherapy (HIPEC) as a new innovative therapeutic option for selected patients with peritoneal carcinomatosis [54,55].

CRS consists of numerous surgical procedures depending on the extent of peritoneal tumour manifestation. Surgery may include parietal and visceral peritonectomy, greater omentectomy, splenectomy, cholecystectomy, resection of liver capsule, small bowel resection, colonic and rectal resection, (subtotal) gastrectomy, lesser omentectomy, pancreatic resection, hysterectomy, cholecystectomy, ovariectomy, urinary bladder resection, and Hudson procedure (en-bloc removal of uterus, ovaries, pouch of Douglas, peritoneum, recto-sigmoid). In patients with mucinous tumours and infiltration of the umbilicus, an omphalectomy is necessary [56,57].

The residual disease is classified intraoperatively using the completeness of cytoreduction (CCR) score.

CCR-0 indicates no visible residual tumor and CCR-1 residual tumor nodules ≤ 2.5 mm. CCR-2 and CCR-3 indicate residual tumor nodules between 2.5 mm and 2.5 cm and > 2.5 cm, respectively [58].

### Hipec

In vitro studies could show that hyperthermia may potentiate the cytostatic effects. Moreover, hyperthermia leads to direct cytotoxic effects such as protein denaturation, induction of apoptosis and inhibition of angiogenesis [55]. For the performance of HIPEC one inflow and three outflow drainages are placed subphrenically and in the small pelvis. The cytostatic agent is infused via the inflow drainage. The intra- peritoneal temperature is monitored by two sensors placed in the inflow catheter and in the Douglas pouch. The intraperitoneal temperature should reach 41–42°C. The perfusion time ranges from 30 to 120 minutes depending on the protocol and the drug used. HIPEC can be performed in open or closed abdomen technique. There seem to be no significant differences between the two techniques regarding morbidity and mortality rates as well as patient survival [58].

Recent developments include the use of CO2 recirculation and laparoscopy assisted HIPEC.

### Complications

In a systematic review of 24 studies (2787 patients) performed in 2009 by Chua et al. [59], the overall mean mortality rate after CRS and HIPEC was 2.9%. The range of major or grade III or IV morbidity was 0–52%.

Small bowel perforations and anastomotic leaks are the most common and clinically significant GI complications after CRS and HIPEC. Preventive measures such as preservation of the gastroepiploic arcade and prophylactic suture of the greater gastric curvature can reduce the risk of gastric perforation [57].

Other complications include surgery related complications like intra-peritoneal abscesses, pancreatic fistulas, biliary fistulas, chyle leak, prolonged ileus, gastric stasis, venous thromboembolism, urinary tract and vascular access infections, pleural effusion, pneumonia and chemotherapy related complications like neutropenia, anemia, thrombocytopenia, heart, liver, and renal toxicity [60].

## Future Trends and Future Perspectives

### Ozone Therapy

Ozone possesses direct cytotoxic activity on tumour cells and also activates immune system to kill cancer cells along with activation and up-regulation of antioxidant enzymes. Intraperitoneal ozone therapy results in local oxidant activity without multi-organ toxicity. In a study conducted by Bocci et al., patients with advanced-stage peritoneal carcinomatosis who underwent intraperitoneal ozone therapy showed prolonged survival [61].

### Photodynamic Diagnosis (PDD) and Photodynamic Therapy (PDT)

In photodynamic diagnosis, photosensitizers like 5-aminolevulinic acid and its derivatives are used. 5-ALA is taken up specifically by cancer cells which express β-amino acid transporter. 5-ALA is converted into protoporphyrin IX which on exposure to light exhibits fluorescence. These fluorescent properties are highly useful for the identification of cancer tissues during surgery and facilitate the accurate and more comprehensive fluorescence-guided resection of cancer tissues. PDD has been proved to be more sensitive in detecting metastases in gastric [62] and pancreatic cancers. As a therapeutic modality, PDT uses the ability of light-excited photosensitizers to produce high levels of reactive oxygen species [63].

### Intraperitoneal Pretargeted Radioimmunotherapy (PRIT)

RIT offers parenteral administration of therapeutic radiation with highly specific anti-tumour antibodies, making it well-suited for treatment of PC [64]. In RIT, the tumour is pretargeted with non-radioactive antibody, followed with separate administration of the radioactive payload. For example, in a study undertaken by Chandler et al. [65]; for treatment of GPA 33 expressing colorectal PC, a high-affinity anti-GPA33/anti-DOTA bispecific antibody (BsAb) is administered, followed by clearing agent (i.v.), and lutetium-177 (Lu-177) or yttrium-86 (Y-86) radiolabelled DOTA-radiohapten (i.p.) for beta/gamma-emitter therapy and PET imaging, respectively.

In their study, single-cycle treatment significantly prolonged median survival approximately two-fold in comparison with controls (P = 0.007). With three-cycle therapy, 75% survived long-term (MS > 183 d).

### Radiotherapy with Radio Labelled Homing Peptides

Alpha-particles kill cells due to induction of double strand breaks in DNA with a high relative biological effectiveness. As the range of the particles is only 28-100 mm in mammalian tissues, the development of carriers mediating specific uptake into the nucleus of tumour- or tumour endothelial cells is important to optimize therapeutic efficacy. Due to its high linear energy transfer, short half-life, and uncomplicated use, 213Bi is promising for medical applications which release alpha particles. F3 (vascular tumour homing peptide F3) is internalized into the nucleus of tumour cells and tumour endothelial cells in vitro and in vivo [66]. DTPA chelates Bi and linked to F3 dimer (DTPA-[F3]_2_). In a study undertaken by Drecoll et al. [67], the mean survival of mice was 51 and 53 days for mice treated with 213Bi-DTPA or PBS, whereas mice treated with 213Bi-DTPA-[F3]2 lived an average of 93.5 days, indicating a significant (80%; p,0.001) increase of survival by 213Bi-DTPA-[F3]2.

## Mimics of Peritoneal Carcinomatosis

### Pseudomyxoma Peritonei

Pseudomyxoma peritonei is characterised by spread of thick mucinous material within the peritoneal cavity due to rupture of mucinous neoplasms [68]. It most commonly occurs due to benign mucinous cystadenoma/mucinous adenocarcinoma of the appendix and mucinous tumours of the ovaries. Radiologically, it is characterised by presence of loculated ascites, multiple low-attenuation nodules with or without curvilinear calcification [69]. Scalloping of the surfaces of intraperitoneal organs like the liver and spleen is highly characteristic of pseudomyxoma peritonei [70].

### Peritoneal Lymphomatosis (PL)

Most peritoneal lymphomatosis are non-Hodgkin Lymphoma type. Peritoneum is mostly secondarily involved by pre-existing lymphoma elsewhere with primary peritoneal lymphomatosis a rare occurrence [68]. On CT, it is characterised by the presence of peritoneal thickening, peritoneal nodules or masses, omental caking, mesenteric nodularity and haziness along with extensive lymphadenopathy and hepatosplenomegaly [71,72]. Presence of concomitant organomegaly and lymphadenopathy helps to differentiate PL from PC [68].

### Malignant Peritoneal Mesothelioma

Malignant mesothelioma is an uncommon neoplasm originating from mesothelial cells or mesenchymal cells of pleura, peritoneum, or pericardium [73]. Radiologically, it has been classified into two types: dry and wet types. Wet type of peritoneal mesothelioma is characterised by extensive ascites, diffuse thickening of parietal and visceral peritoneum encasing bowel loops, omental thickening and caking along with mesenteric leaves thickening and fat stranding. In the dry type, the soft tissue mass is usually confined to single quadrant of abdomen with no or minimal ascites. The amount of ascites in peritoneal mesothelioma is very small as compared to the soft tissue mass/peritoneal thickening of peritoneal carcinomatosis. It is difficult to differentiate peritoneal mesothelioma from PC radiologically; however, history of asbestosis exposure, presence of pleural plaques, and relatively lesser amount of ascites are some features favouring malignant mesothelioma over PC [74,75].

### Diffuse Peritoneal Leiomyomatosis (DPL)

This condition is characterised by presence of multiple leiomyomas within peritoneal cavity. It usually occurs in female with history of Cesarean section/hysterectomy or myomectomy [4]. Radiologically appears as multiple smooth marginated soft tissue masses showing delayed enhancement within peritoneal cavity with no ascites, peritoneal thickening or omental haziness [6]. Presence of typical imaging findings, history of surgery or presence of uterine leiomyomas are clues for differentiation of DPL from PC [4].

### Tuberculous Peritonitis

Spread of tuberculous infection into the peritoneal cavity can occur via hematogenous, lymphatic or direct spread [18]. Tuberculous peritonitis is divided into three types: dry, wet, and fibrotic-fixed types.

In the wet type, ascites along with peritoneal thickening, necrotic mesenteric lymphadenopathy is seen. Fibrotic fixed type is characterised by large omental masses with matted and tethered bowel loops and while in dry type, caseous nodules, fibrotic peritoneal reaction and adhesions are seen with no ascites.

## Conclusion

Management of peritoneal carcinomatosis has evolved significantly in recent years. Radiologists play a pivotal role in the selection of patients who can benefit from CRS with HIPEC/EPIC, which prolong survival in these patients. Incorrect selection of patients for surgery can result in increased morbidity and mortality in unsuitable candidates.
